# Corrigendum to “Incidence of Calf Morbidity and Its Predictors in North Shewa, Amhara, Ethiopia”

**DOI:** 10.1155/2020/2095278

**Published:** 2020-09-07

**Authors:** Rahma Mohammed, Hailemariam Kefyalew, Dawit Kassaye

**Affiliations:** College of Veterinary Medicine, Haramaya University, Dire Dawa, Ethiopia

In the article titled “Incidence of Calf Morbidity and Its Predictors in North Shewa, Amhara, Ethiopia” [1], the acknowledgements should read as follows:

We would like to extend our deepest appreciation to our study participants for their willingness to participate in the study and allowing us to conduct our study on their calves' management system. We are thankful to Professor Alemayehu Lemma from Addis Ababa University, College of Veterinary Medicine and Agriculture, for his financial support and advice. We are also grateful to the National Animal Health Diagnostic and Investigation Center (NAHDIC) located at Sebeta, Ethiopia, for the support given to us to undertake the full laboratory work.
